# Pharmacokinetic study of single and multiple oral administration of glutamine in healthy Beagles

**DOI:** 10.3389/fphar.2022.1014474

**Published:** 2022-09-30

**Authors:** Fanxi Guo, Dongying Liu, Yuqing Zhou, Yuanqian Yu, Yidan Xu, Yuanpeng Zou, Chongyang Li, Fengyichi Zhang, Zugong Yu

**Affiliations:** Laboratory of Veterinary Pharmacology and Toxicology, College of Veterinary Medicine, Nanjing Agricultural University, Nanjing, China

**Keywords:** glutamine tablets, pharmacokinetics, multiple administration, single administration, Beagles

## Abstract

Glutamine is an amino acid that is mainly used for the treatment of gastrointestinal diseases in clinic, but there is a lack of such medicine in veterinary clinic, and its research in dogs has never been seen. This study aimed to investigate the pharmacokinetics of single and multiple administration of glutamine (Gln) tablets in Beagles. Twenty-four healthy Beagles were randomly selected for the pharmacokinetic study of a single dose of low (120 mg/kg), medium (240 mg/kg), and high (360 mg/kg) Gln tablets. After 7 days of washout period, six Beagles in the medium group were selected for a multiple-dose pharmacokinetic study, 240 mg/kg twice a day for 7 days. The Gln concentration in plasma was determined by a validated UPLC-MS/MS method. The results of single oral administration of different doses of Gln tablets showed that C_max_, AUC_0→t_, AUC_0→∝_ had a certain linear relationship with the dosage. T-tests were performed for single and multiple administration of T_max_, C_max_, t_1/2λz_, AUC_0→t_, and AUC_0→∝_, and the results showed no significant differences (*p* > 0.05). Therefore, Gln tablets were absorbed quickly by oral administration, and there was no accumulation in Beagles after 7 days of administration.

## 1 Introduction

Gastrointestinal mucosal barrier injury is closely related to gastrointestinal diseases (Turner 2009; Camilleri et al., 2012), and increasing evidence shows that the increase of intestinal permeability plays a major pathogenic role in some intestinal diseases (Zuckerman et al., 1993; Spiller et al., 2000; Marshall et al., 2004; Dunlop et al., 2006). Vomiting and diarrhea caused by gastrointestinal mucosa injury are common in pets (Niu et al., 2021), but the lack of medicine that can effectively repair the intestinal mucosa leads to slow recovery, which affects the health and quality of life of pets. Therefore, medicines that can nourish and repair intestinal mucosal cells are urgently needed in veterinary clinic.

Glutamine (Gln) is an amino acid whose main function is to provide nutrition to gastrointestinal cells and immune cells and maintain the integrity of gastrointestinal mucosa (Ardawi and Newsholme 1983; Burke et al., 1989; Jiang and Fang 2009; Wang and Liu 2009). Therefore, Gln is also suitable for the treatment of diseases related to gastrointestinal mucosal injury in pets. Gln has been widely used in human clinical medicine in a variety of dosage forms, mainly for the treatment of gastritis, gastric ulcer and duodenal ulcer (Griffiths et al., 2000; Anderson and Lalla 2020). A large number of clinical controlled studies have proved the good efficacy of Gln on gastrointestinal diseases (Mochiki et al., 2011; Dai and Meng 2015; Zuo 2015; Yao et al., 2016; Zhou et al., 2019).

Pharmacokinetic studies of Gln in humans (Ziegler et al., 1990; Ward et al., 2003; Zhu et al., 2003; Berg et al., 2005; Tang et al., 2005; Yu et al., 2014; Du et al., 2015; Han et al., 2016; Morris et al., 2022) and rats (Wang et al., 2015) have been reported in some literatures and the results show that oral Gln was rapidly absorbed and metabolized *in vivo*. However, there was a lack of relevant studies in pets, so pharmacokinetic studies of Gln in dogs are necessary to introduce it into pet clinical practice.

The aim of this study was to clarify the absorption, distribution, metabolism and excretion of Gln tablets in dogs, provide theoretical basis for the introduction of Gln tablets into pet clinical practice, and provide reliable data for the formulation of clinical recommended drug administration program. The pharmacokinetic study of single and multiple oral administration of Gln tablets in dogs was conducted to study the correlation between main pharmacokinetic parameters and dosage, and to compare the similarities and differences between single and multiple administration to investigate the safety of multiple administration.

## 2 Materials and methods

### 2.1 Animals

Twenty-four healthy Beagles (half male and half female) aged from 15 to 17 months, weighing 7.0–10.9 kg, were purchased from Jiangsu Yadong Experimental Animal Research Center. The laboratory animal production license number: SCXK (Su) 2016-0009. All dogs were kept in individual cages and given a daily ration of drug-free feed to maintain proper weight and adequate water. Before each experiment, dogs were fasted for 12 h and water was forbidden for 1 h. In addition, 5 ml of water was fed with a syringe during drug administration, water was fed for 2 h and food for 4 h after drug administration. All animal experiments were conducted in accordance with the guidelines of the Animal Ethics Committee of Nanjing Agricultural University (Nanjing, China).

### 2.2 Drugs and reagents

Gln tablets were produced by Nanjing Jindun Animal Pharmaceutical Co., Ltd., and the production batch number was 20190501. The working standard of Gln and Penciclovir (Internal Standard) were obtained from China National Institutes for Drug Control. Acetonitrile and formic acid were HPLC grade, and ammonium formate was LC/MS grade.

### 2.3 Experimental design

#### 2.3.1 Pharmacokinetic study design of single dose

Pharmacokinetics of low (120 mg/kg), medium (240 mg/kg) and high (360 mg/kg) doses of Gln tablets were studied in 24 healthy Beagles, including 6 in the low and high dose groups and 12 in the medium dose group. All animals were weighed and blank plasma samples were collected at −24, −18, −12, and 0 h before administration. The mean value of Gln concentration in blank plasma was used as baseline concentration for correction. Plasma samples were collected at 0.17, 0.33, 0.5, 0.67, 0.83, 1, 1.25, 1.5, 1.75, 2, 2.5, 3, 4, 6, 8, 10, 12, and 24 h after oral administration, and the Gln concentration in plasma was measured by UPLC-MS/MS. The concentration of drug-derived Gln was obtained by subtracting the baseline concentration before administration from the total Gln concentration in plasma.

#### 2.3.2 Pharmacokinetic study design of multiple dosing

Six Beagles (half males and half females) from the medium-dose group were selected for a pharmacokinetic study of multiple doses (240 mg/kg) after a 1-week cleaning period following a single dose. Multiple oral pharmacokinetic studies were administered twice a day, with an interval of 12 h, for 7 consecutive days (13 times). Blank blood samples were collected at 24, 18, 12, and 0 h before the first dose. The mean glutamine concentration in blank blood samples collected at these times served as the baseline endogenous glutamine concentration. Blood samples were collected at the following time points after the start of dosing: before the 7th dosing (the morning of the 4th day of dosing), before the 9th dosing (the morning of the 5th day of dosing), before the 11th dosing (The morning of the 6th day of administration), 6 h after the 11th administration, before the 12th administration (the evening of the 6th day of administration), before the 13th administration (the morning of the 7th day of administration), and 10, 20, 30, 40, 50 min, 1, 1.25, 1.5, 1.75, 2, 2.5, 3, 4, 6, 8, 10, 12, 24 h after the 13th administration. The Gln concentration in plasma was measured by UPLC-MS/MS, and the concentration of drug-derived Gln was obtained by subtracting the baseline concentration before first administration from the total Gln concentration in plasma.

#### 2.3.3 Collection and storage of samples

About 3–3.5 ml of blood is collected from the dog’s forelimb vein with a disposable vacuum blood collection tube using heparin (dry powder) anticoagulation. The blood samples were centrifuged at 2,152 ± 5 g for 10 min at 4°C, and the separated plasma was divided into 0.5 ml/part. All blood samples should be centrifuged and aliquoted within 60 min after collection, and the aliquoted plasma should be immediately stored in an ultra-low temperature refrigerator (−80 ± 10°C).

### 2.4 Analytical method

The measurement of plasma FFC levels was performed using a ultra performance liquid chromatography/tandem mass spectrometry (UPLC-MS/MS) device (Waters XEVO TQD, Waters Technologies Co. Ltd., United States) comprised a UPLC separation system and a triple quadrupole mass analyzer.

Separation was carried out using a Waters Acquity UPLC BEH Amide column (100 mm╳2.0 mm, particle size 1.7 µm; Waters Technologies Co., Ltd., Milford United States). The injection volume was 5 µl. The temperature of the column and auto-sampler was set at 35 and 6°C, respectively. The mobile phase was composed of acetonitrile (mobile phase A) and 40 mmol/L ammonium formate mixture (pH = 3.0 with formic acid, mobile phase B) at a flow rate of 0.4 ml/min. The gradient elution was shown in Table 1. This resulted in an overall run time of 5.5 min.

**TABLE 1:** The gradient elution.

Time, min	Acetonitrile, %	40 mmol/L ammonium formate mixture
0.0	80.0	20.0
0.5	80.0	20.0
3.0	70.0	30.0
4.0	70.0	30.0
4.1	80.0	20.0
5.5	80.0	20.0

Detection was performed using a TQD triple quadrupole mass spectrometer equipped with an electrospray ionization source in the electrospray positive ion mode (ESI + ). Nitrogen was used as both drying and nebulizing gas. Product ions were detected using the multiple reaction-monitoring (MRM) mode, using argon as collision gas. The capillary voltage and source temperature were optimized at 3.0 kV and 150°C, respectively. The collision energy and cone voltage were optimized for each compound individually. The collision energy varied from 10 to 35 eV, and the cone voltage varied from 23 to 36 V. The MS/MS transition compounds for Gln were 147.2 > 84 and 147.2 > 130 at collision voltages of 10 and 18 eV, respectively. And for Penciclovir (Internal standards) were 254.1 > 135 and 254.1 > 152 at collision voltages of 35 and 18 eV (Liu et al., 2022). Data were collected and processed using the MassLynx software 4.1.

### 2.5 Sample preparation

The plasma pretreatment method was acetonitrile and ammonium formate solution for protein precipitation, and penciclovir was used as the internal standard. 100 μl of plasma samples were thawed at room temperature, added 20 μl of water, 200 μl of IS working solution, and 800 μl of initial mobile phase, then vortexed for 2 min and centrifuged at 4°C 14,462 g for 10 min. 780 μl initial mobile phase was added to 20 μl of supernatant liquid, then vortexed for 1 min. Ultimately, the samples were filtered through membrane filters (0.22 μm). The processed samples were injected into UPLC-MS/MS for analysis.

### 2.6 Pharmacokinetic and statistical analysis

Pharmacokinetic parameters were determined with Phoenix WinNonlin Professional software (Version 8.1) by using non-compartmental analysis. The area under the plasma concentration-time curve (AUC) was calculated by the linear trapezoidal linear interpolation method, and the pharmacokinetic parameters such as the terminal elimination half-life (t_1/2λz_), the maximum concentration of Gln in plasma (C_max_), the time to reach C_max_ (T_max_) were also obtained. All pharmacokinetic results were presented as mean ± SD.

## 3 Results

### 3.1 Results of a single oral glutamine tablets at different doses

**FIGURE 1:**
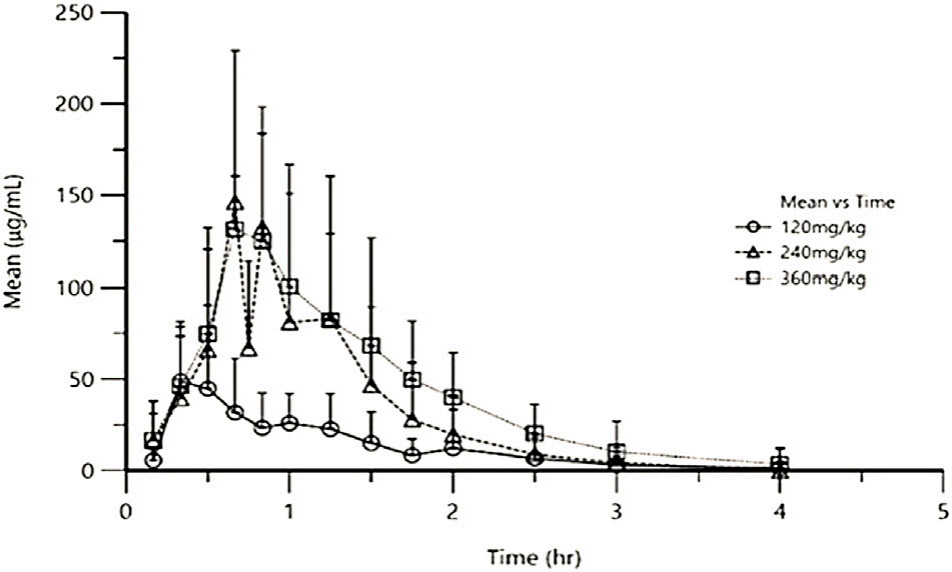
Mean exogenous Gln concentration-time curves in Beagles’ plasma after single oral administration of three different doses of Gln tablets.

After a single oral dose of low-dose (120 mg/kg), medium-dose (240 mg/kg), and high-dose (360 mg/kg) in Beagles, the mean plasma concentration-time curve of exogenous Gln was shown in Figure 1 and the mean pharmacokinetic parameters (mean ± SD) were shown in Table 2.

**TABLE 2:** Mean pharmacokinetic parameters of exogenous Gln in Beagle’ plasma after single oral administration of three different doses of Gln tablets. (mean ± SD).

Parameter	Units	120 mg/kg (*n* = 6)	240 mg/kg (*n* = 12)	360 mg/kg (*n* = 6)
λ_z_	1/h	2.35 ± 1.98	2.38 ± 1.57	1.83 ± 0.89
t_1/2λz_	h	0.49 ± 0.33	0.42 ± 0.27	0.58 ± 0.56
T_max_	h	0.93 ± 0.63	0.85 ± 0.29	1.00 ± 0.76
C_max_	μg/mL	64.56 ± 28.67	136.11 ± 72.51	141.41 ± 60.65
AUC_0→t_	h·μg/mL	52.63 ± 19.85	116.30 ± 75.15	160.15 ± 51.84
AUC_0→∝_	h·μg/mL	58.73 ± 20.20	138.76 ± 87.45	191.85 ± 30.37
MRT	h	1.13 ± 0.62	1.03 ± 0.33	1.33 ± 0.37

λ_z_, terminal phase rate constant; t_1/2λz_, terminal elimination half-life; T_max_, time needed to reach C_max_; C_max_, peak plasma concentration; AUC_0–t_, the mean area under the concentration–time curve from 0 h to last time collected samples; AUC_0–∝_, the mean area under the concentration–time curve from 0 h to infinity; MRT, mean residence time.

### 3.2 Results of a dose-dependent analysis of a single oral dose of glutamine tablets

**FIGURE 2:**
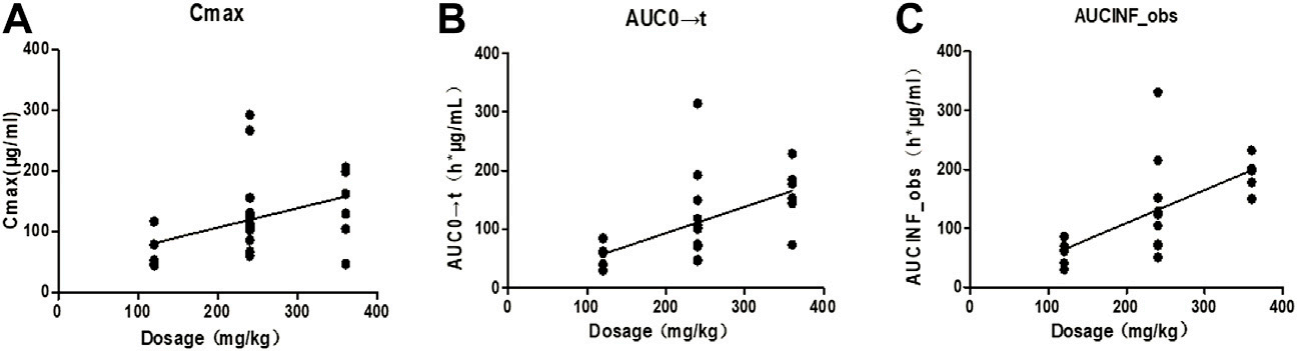
Correlation curves of pharmacokinetic parameters and dosage of each Beagle. Note: **(A)**. The correlation curve of Cmax/dose of each Beagle; **(B)**. The correlation curve of AUC0→t/dose of each Beagle; **(C)**. The correlation curve of AUC0→∝/dose of each Beagle.

Taking the dose as the abscissa, and the C_max_, AUC_0→t_ and AUC_0→∝_ of each tested Beagle as the ordinate, the linear regression was performed. The results were shown in Figure 2. After Spearman rank correlation analysis, the results were shown in Table 3. The results showed that the correlation coefficient r of C_max_-dose was 0.5108 (*p* < 0.05), the correlation coefficient r of AUC_0→t_-dose was 0.6725 (*p* < 0.01), and the correlation coefficient r of AUC_0→∝_-dose was 0.7463(*p*<0.01). It showed that C_max_, AUC_0→t_, AUC_0→∝_ were positively correlated with the dose, respectively.

**TABLE 3:** Results of ANOVA for the main pharmacokinetic parameters-dosage of three different dosage groups.

Parameter	Spearman’s rank correlation coefficient r	*P*
C_max_	0.5108	<0.05
AUC_0→t_	0.6725	<0.01
AUC_0→∝_	0.7463	<0.01

**FIGURE 3:**
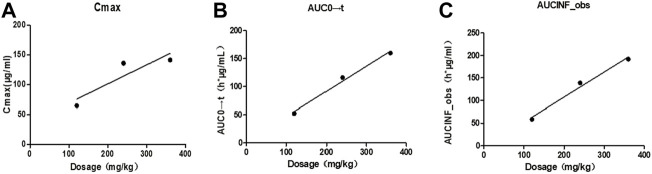
Correlation curves of mean pharmacokinetic parameters and dosage of Beagles. Note: **(A)**. The correlation curve of mean Cmax/dose of Beagles; **(B)**. The correlation curve of mean AUC0→t/dose of Beagles; **(C)**. The correlation curve of mean AUC0→∝/dose of Beagles.

Taking the dose as the abscissa and taking the mean of each C_max_, AUC_0→t_ and AUC_0→∝_ as the ordinate, the linear regression was performed. The results were shown in Figure 3, and the linear regression equation and determination coefficient *r* ^*2*^ were shown in Table 4.

**TABLE 4:** Linear regression results of the means of the main pharmacokinetic parameters for three dosage groups.

Parameter	Regression equation	*r* ^2^
C_max_	*Y* = 0.3202x + 37.1730	0.8014
AUC_0→t_	*Y* = 0.4480x + 2.1700	0.9888
AUC_0→∝_	*Y* = 0.5547x - 3.3368	0.9865

### 3.3 Results of a comparative pharmacokinetic study of single and multiple oral glutamine tablets

**FIGURE 4:**
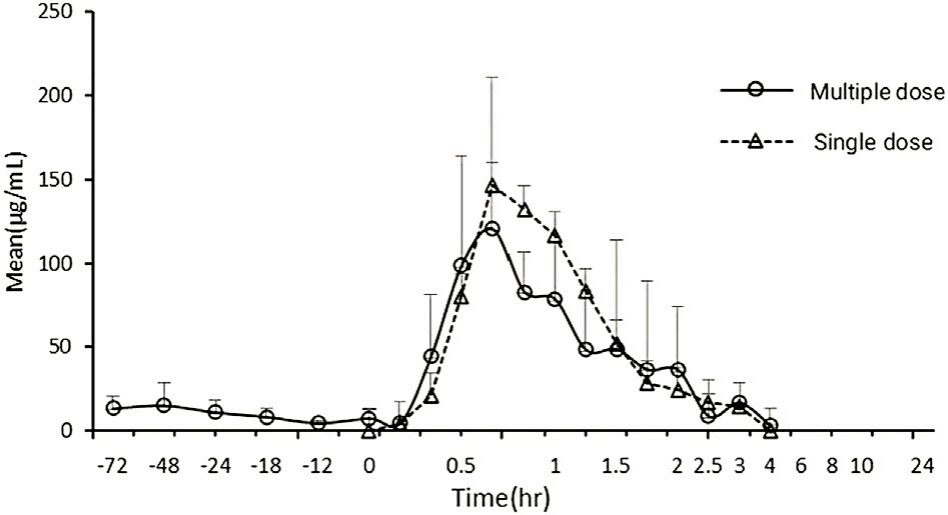
Mean exogenous Gln concentration–time curves in Beagles’ plasma after single and multiple oral administration of 240 mg/kg Gln tablets (mean ± SD, *n* = 6).

The average pharmacokinetic parameters of 240 mg/kg Gln tablets after single or multiple oral administration of 6 Beagles were shown in Table 5, and the mean Gln concentration in plasma versus time curve was shown in Figure 4. SPSS software was used to conduct *t*-test on the pharmacokinetic parameters of single and multiple administrations. The results showed that there was no significant difference compared with single administration (*p* > 0.05).

**TABLE 5:** Comparison of the mean pharmacokinetic parameters of exogenous Gln between single and multiple oral administration of 240 mg/kg Gln tablets in Beagls (mean ± SD, *n* = 6).

Parameter	Units	Single dose	Multiple doses
t_1/2λz_	h	0.51 ± 0.28	0.27 ± 0.15
T_max_	h	0.81 ± 0.16	0.89 ± 0.36
C_max_	μg/mL	176.21 ± 82.65	150.52 ± 66.88
AUC_0→t_	h·μg/mL	155.36 ± 88.22	104.05 ± 35.66
AUC_0→∝_	h·μg/mL	167.40 ± 93.34	107.64 ± 32.62
MRT	h	1.26 ± 0.36	1.09 ± 0.40

## 4 Discussion

As an endogenous substance, the pharmacokinetics of glutamine may be affected by the level of endogenous substances, food, and rhythm changes compared with other drugs (Marzo et al., 1993; Dissanayake 2010; Zhang et al., 2011). This study optimized the experimental design by adjusting for baseline substance levels, administering supra-therapeutic doses, and standardizing dietary management. The methods reported in the literature for the base value subtraction of endogenous substances mainly include the mean value subtraction method (Wang et al., 2015) and the point-to-point subtraction method (Berg et al., 2005; Han et al., 2016). Previous studies by our group (Unpublished prior to submission of this article, but accepted.) showed that the concentration of endogenous Gln in the plasma of Beagles remained basically constant within 48 h, with no circadian rhythm and periodic differences.

Based on the stable endogenous glutamine concentration of beagle dogs, and referring to the guiding principles for human bioavailability and bioequivalence test of pharmaceutical preparations (guiding principles 9,011, Part IV, Chinese Pharmacopoeia, 2020 Edition), blank blood samples were collected at four time points of 24, 18, 12, and 0 h before administration in this study, and the endogenous glutamine concentration was measured, and the average value was calculated as the baseline concentration. The exogenous glutamine concentration was obtained by subtracting the baseline concentration from the total glutamine concentration in blood after administration, and the pharmacokinetics was analyzed with the exogenous glutamine concentration.

In this study, the pharmacokinetics of Gln tablets were tested in dogs with a single oral administration of three dosages, and according to the analysis of exogenous Gln concentration in plasma it can be seen that: after oral administration of glutamine tablets, T_max_ was 30–60 min, C_max_ were 64.56 ± 28.67 μg/ml (120 mg/kg), 136.11 ± 72.51 μg/ml (240 mg/kg) and 141.41 ± 60.65 μg/ml (360 mg/kg), respectively, and then gradually decreased to the normal range within 90–120 min (120 mg/kg) or 120–240 min (240 mg/kg and 360 mg/kg). According to the report of Ziegler (Ziegler et al., 1990), after oral administration of glutamine 100 mg/kg and300 mg/kg in healthy people, the T_max_ was 30–45 min, and the C_max_ were 44.89 μg/ml (100 mg/kg) and 88.71 μg/ml (300 mg/kg). And then dropped steadily to the normal range within 90–120 min (100 mg/kg) or 180–240 min (300 mg/kg). From the above results, it can be seen that under the condition of oral low-dose glutamine, the pharmacokinetic behavior in dogs was more similar to that in humans, and the drug-induced C_max_ in dogs at high doses was higher than that in humans.

The mean values of C_max_ (*r* ^2^ = 0.8014), AUC_0→t_ (*r* ^2^ = 0.9888), AU_C0→∝_ (*r* ^2^ = 0.9865) showed a certain linear relationship with the administered dose in this test. It was consistent with the results reported by Ziegler that the concentration of glutamine in blood increased in a dose-related after oral glutamine of low and high doses in healthy people (Ziegler et al., 1990).

When glutamine plays a clinical therapeutic role, it is usually administered multiple times (Rotovnik et al., 2017; Arutla et al., 2019; Chang et al., 2019; Zhou et al., 2019). Therefore, it is necessary to clarify the pharmacokinetic characteristics of Gln tablets when they are administered multiple times in dogs, and to investigate whether it will cause accumulated toxicity. The result of the multiple-dose study showed a mean Gln concentration of 8.97 μg/ml at steady state after administration (baseline correction), with an accumulation factor of 1.00. The pharmacokinetic parameters of six dogs who received both single and multiple doses were tested by *t* test. The results showed that there was no significant difference in T_max_, C_max_, t_1/2λz_, AUC_0→t_ and AUC_0→∝_ for single and multiple administrations (*p* > 0.05). It showed that the absorption, distribution and metabolism of Gln tablets *in vivo* after repeated administration were similar to those of single administration, and there was no accumulation *in vivo*.

The results of this study showed that after oral administration of glutamine tablets in dogs, the plasma concentration generally reached a peak value at 30–60 min after administration, with an average peak concentration of 64.56 ± 28.67 μg ml^−1^ (120 mg kg^−1^), 136.11 ± 72.51 μg ml^−1^ (240 mg kg^−1^) and 141.41 ± 60.65 μg ml^−1^ (360 mg kg^−1^), then gradually decreased to the normal range within 90–120 min (120 mg kg^−1^) or 120–240 min (240 mg kg^−1^ and 360 mg kg^−1^).

It has been reported that after oral administration of glutamine 0.1 g/kg (low dose) and 0.3 g/kg (high dose) in adults, the blood concentration of glutamine increases in proportion to the dose. The peak appeared at 30–45 min after the intake of glutamine, and then steadily decreased to the normal range within 90–120 min (low dose) or 180–240 min (high dose). After administration of 0.1 g kg^−1^ (low dose) and 0.3 g kg^−1^ (high dose) glutamine, the peak blood concentration of glutamine was 1,028 ± 97 μmol L^−1^ (150.24 μg ml^−1^) and 1,328 ± 99 μmol L^−1^ (194.09 μg ml^−1^) respectively. The above shows that the pharmacokinetic behavior of glutamine in dogs is similar to that in humans after oral administration.

The dosage regimen of glutamine in the treatment of human gastroenteritis and gastric ulcer is: oral, 0.5 g each time, 2–3 times a day. The body surface area calculation method could be used to convert the human dose to the dog dose, and the dog’s dose was 30–60 mg/kg BW, treatment is repeated at 8 h or 12 h intervals.

Based on available pharmacokinetic data, we speculate that the human dose can be converted into the dog dose for further clinical trials. But the formulation of the final dosing regimen for dogs needs to be based on the results of further clinical trials.

## 5 Conclusion

The results of single dose showed that C_max_, AUC_0→t_, AUC_0→∝_ had a certain linear relationship with dosage. There were no significant differences in T_max_, C_max_, t_1/2λz_, AUC_0→t_ and AUC_0→∝_ between multiple administration and single administration (*p* > 0.05), indicating no accumulation of oral Gln in the body.

## Data Availability

The raw data supporting the conclusion of this article will be made available by the authors, without undue reservation.
